# Radiation and Stemness Phenotype May Influence Individual Breast Cancer Outcomes: The Crucial Role of MMPs and Microenvironment

**DOI:** 10.3390/cancers11111781

**Published:** 2019-11-12

**Authors:** María Auxiliadora Olivares-Urbano, Carmen Griñán-Lisón, Sandra Ríos-Arrabal, Francisco Artacho-Cordón, Ana Isabel Torralbo, Elena López-Ruiz, Juan Antonio Marchal, María Isabel Núñez

**Affiliations:** 1 Department of Radiology and Physical Medicine, University of Granada, 18016 Granada, Spain; auxi_ou@hotmail.com (M.A.O.-U.); sandrariosarrabal@hotmail.com (S.R.-A.); fartacho@ugr.es (F.A.-C.); anatorralboromero@gmail.com (A.I.T.); 2 Biopathology and Regenerative Medicine Institute (IBIMER), Centre for Biomedical Research, University of Granada, 18100 Granada, Spain; glcarmex@gmail.com; 3 Department of Human Anatomy and Embryology, Faculty of Medicine, University of Granada, 18016 Granada, Spain; 4 Biosanitary Research Institute, ibs.Granada, 18012 Granada, Spain; 5 Department of Health Sciences, University of Jaén, E-23071 Jaén, Spain; elenalopru@gmail.com; 6 CIBER Epidemiology and Public Health (CIBERESP), 28029 Madrid, Spain

**Keywords:** breast cancer outcomes, radiotherapy, cancer stem cells, stemness phenotype, irradiation, 3D culture, matrix metalloproteases, inhibitors, targeted treatment, Matrigel

## Abstract

Breast cancer is the most common cancer in women. Radiotherapy (RT) is one of the mainstay treatments for cancer but in some cases is not effective. Cancer stem cells (CSCs) within the tumor can be responsible for recurrence and metastasis after RT. Matrix metalloproteases (MMPs), regulated mainly by tissue inhibitors of metalloproteinases (TIMPs) and histone deacetylases (HDACs), may also contribute to tumor development by modifying its activity after RT. The aim of this work was to study the effects of RT on the expression of MMPs, TIMPs and HDACs on different cell subpopulations in MCF-7, MDA-MB-231 and SK-BR-3 cell lines. We assessed the in vitro expression of these genes in different 3D culture models and induced tumors in female NSG mice by orthotopic xenotransplants. Our results showed that gene expression is related to the cell subpopulation studied, the culture model used and the single radiation dose administered. Moreover, the crucial role played by the microenvironment in terms of cell interactions and CSC plasticity in tumor growth and RT outcome is also shown, supporting the use of higher doses (6 Gy) to achieve better control of tumor development.

## 1. Introduction

Breast cancer (BC) is the most common cancer in women and ranks second worldwide, contributing to 11.6% of all new cases. Nevertheless, BC ranks fifth for mortality worldwide, contributing to 6.6% of the total cancer deaths [1]. BC is a complex disease with high inter- and intra-tumor heterogeneity [2]. Tumor subtype and resistance to treatment are involved in BC development and growth as well as cancer stem cells (CSCs), a small portion of cells found within tumors [3].

CSCs were first reported in 1996 based on identical chromosomal alterations in contiguous regions of the mammary epithelium [4,5,6]. The concept of CSCs has been around for decades and is still evolving [2]. There are several theories for the origin of CSCs, but the dynamic model is the best supported from the field of research [7,8]. This theory emphasizes the relevance of the dynamic and changing nature of cancer and the CSC population in which the tumour microenvironment plays a crucial role in determining CSC characteristics within a malignancy [8]. CSCs are cells within tumors that exhibit increased tumorigenic potential and certain characteristics of normal stem/progenitor cells [2]. CSCs are a subject of intense research, not only because they are related to tumor initiation, maintenance and progression, but also because of their resistance to radiotherapy (RT) and chemotherapy. For this reason, CSCs play an important role in the processes of metastasis and tumor recurrence [3,9] and, therefore, they are suitable candidates as markers for more accurate diagnosis of metastatic BC, prognosis assessment and therapeutic targeting [10]. For this reason, CSC characterization is paramount in the research to achieve better clinical outcomes. Ginestier et al. found higher activity of aldehyde dehydrogenase 1 (ALDH1) in CSCs from colorectal cancer using the Aldefluor assay [11]. Other established CSC membrane markers are CD44^hi^/CD24^−^, CD133^+^ or CD29^hi^/CD61^+^ [2,12].

RT has remained one of the mainstay treatments for cancer for over 100 years [13]. Different RT regimens are used depending on patient characteristics, threshold dose and tumor location (tissues and organs) [14]. In the clinic, the most commonly applied RT regimen is daily fractions of 2 Gray (Gy), known as conventional RT [12]. Alternatively, hypofractionated RT is being increasingly used, which involves higher daily radiation doses given in a shorter period of treatment [15]; it is increasingly being used due to similar efficacy and lower toxicity than conventional RT [16]. The use of radiation is based on the principle that tumor regions are more sensitive to RT than normal tissues. However, the heterogeneity and the biological complexity of certain tumors result in resistance to RT, where CSCs seem to be involved as well as in metastasis and tumor recurrence after RT [13]. This is due to the capacity of CSCs to overcome the effects of radiation by intrinsic and extrinsic mechanisms, such as DNA repair, cell cycle control points, different survival pathways (Hedgehog, Notch, Wnt/β-catenin), hypoxia and angiogenesis in both the niche and tumor microenvironment [17].

Matrix metalloproteases (MMPs) are a family of enzymes that comprises different subfamilies (collagenases, gelatinases, matrilysins, stromelysins, membrane-type MMPs and other MMPs), which differ in structure, substrate specificity, sequence homology, cellular localization and secretion [18,19]. The main function of MMPs is the remodeling of the extracellular matrix (ECM) [18]. They are also regulators in cell–cell interactions and are involved in the replacement of stromal fibers [20]. Besides, they are also involved in other physiological functions [21] and carcinogenic processes (tumor growth, evasion of apoptosis, angiogenesis, response to inflammation, degradation of collagen in the basement membrane, changes in epithelial-mesenchymal transition (EMT), formation of pre-metastatic niches, invasion and metastasis) [22]. After RT these processes are favored by an increase in the activity of MMPs, which are regulated by endogenous tissue inhibitors (TIMPs) and by epigenetic regulators, such as histone deacetylases (HDACs).

In addition to being endogenous inhibitors of MMPs, TIMPs can also activate pro-MMPs and have activities independent of MMPs, including effects on cell growth and differentiation, angiogenesis, apoptosis and synaptic plasticity [23]. The TIMPs described in humans (TIMP-1 to 4) differ in their inhibition spectrum and in their affinity for human MMPs [24]. HDACs are enzymes that participate in gene silencing by removing acetyl groups from chromatin. However, this deacetylation promotes the evasion of apoptosis and induces a non-response to anti-proliferative signals [25]. Therefore, HDAC inhibitors (HDACIs) are required to compensate for the situation [26]. Changes mainly in MMP expression may contribute to the development of BC and these genes are considered potential prognostic biomarkers for this type of cancer [22]. 

Tissue microenvironment plays a critical role in controlling the form, function, growth and development of tissues. Conventional 2D cell culturing is an easy and efficient technique, but cell growth in monolayer does not mimic well enough the complex cellular microenvironment. To overcome this limitation, the use of 3D cell cultures tries to create in vivo conditions for cellular development and provides an adequate modeling of cancer cell biology. The 3D approach allows crucial cell–cell and cell–ECM interactions, as well as the development of important tissue characteristics. This in turn will allow the production of an adequate structure and the functional differentiation of normal or tumor tissues in vitro. For this reason, 3D cell cultures represent an alternative to animal models [27] and have been chosen for the present research.

The main objective of this work was to study the effects of RT on the expression of MMPs, TIMPs and HDACs. The differences in their expression were analyzed in different culture models and in different cell subpopulations. The results allow us to determine the role of MMPs, TIMPs, HDACs, and CSCs as predictive factors of BC invasion and metastasis.

## 2. Results

### 2.1. Effects of Ionizing Radiation (IR) on CSC Characterization

**Figure 1:**
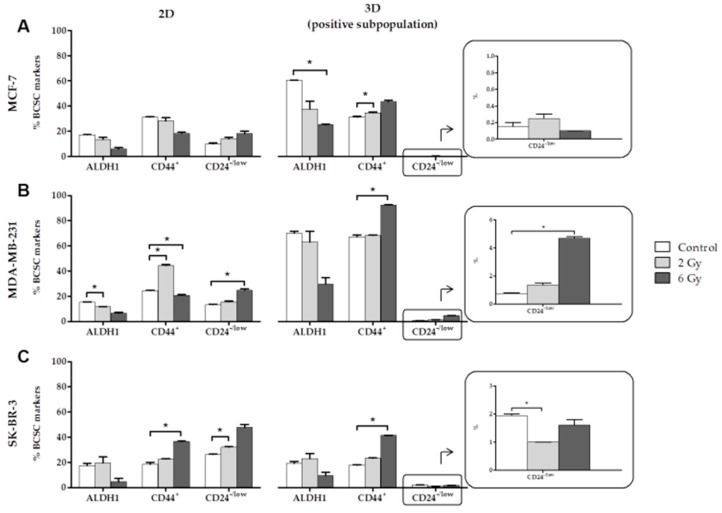
Expression of specific breast cancer stem cells (BCSCs) markers (% of ALDH1, CD44+ and CD24-/low cells) at 0, 2 and 6 Gy IR doses of the MCF-7 (**A**), MDA-MB-231 (**B**) and SK-BR-3 (**C**) cell lines in 2D and 3D cultures. Values are expressed as median ± SEM (error bars) of three independent experiments run in triplicate. Mean value for each experiment was calculated by averaging the triplicates (*n* = 3); significant values are marked with * (comparison of IR doses with non-irradiated control); * *p* < 0.05.

MCF-7, MDA-MB-231 and SK-BR-3 cell lines were grown in a 2D monolayer culture and in a 3D sphere culture in suspension (only the positive subpopulation) and irradiated at 0, 2 and 6 Gy. A total of 24 h post-IR, cells were characterized with specific breast CSCs (BCSCs) markers (ALDH1, CD44^+^ and CD24^−/low^). Figure 1 shows the marker expression for the three cell lines in the two types of cultures (% of ALDH1, CD44^+^ and CD24^−/low^ cells). For the MCF-7 cell line (Figure 1A), the different expression of ALDH and CD44^+^ was associated with the IR dose and the culture conditions. In 2D cultured cells no significant differences in the expression of the markers was found. In 3D cultured cells, the expression of ALDH1 and CD44^+^ was significant when comparing controls vs different doses of radiation as indicated in Figure 1A.

For the MDA-MB-231 cell line (Figure 1B), significant differences were found in the expression of CD44^+^ and CD24^−/low^ in 2D and 3D cultures; however, ALDH1 expression was significant in 2D culture only. After IR, a decrease in ALDH1 and an increase in CD24^−/low^ was detected in the 2D and 3D cultures. However, CD44^+^ showed the highest expression at 2 Gy in 2D cultures and at 6 Gy in 3D cultures. For the SK-BR-3 cell line (Figure 1C), significant differences were found in CD44^+^ expression in both models of cultures when comparing the 6 Gy dose with the control, showing a tendency toward increased expression with higher doses of IR. Significant differences in CD24^−/low^ expression in both cultures were also found when comparing the 2 Gy dose with the control but no relation was found between the increase in IR dose and marker expression. Moreover, to study inherent radioresistance of the generated cell sub-types we measured apoptotic rates 24 h after irradiation in the general subpopulation and ALDH+ subpopulation. Our results showed that the CSC subpopulation in MCF-7 and MDA-MB-231 were more radioresistant (low levels of radio-induced apoptosis) than the general subpopulation (high rate of radio-induced apoptosis) (Figure S2 and Table S8).

### 2.2. Effects of Ionizing Radiation on In Vitro Gene Expression

MCF-7, MDA-MB-231 and SK-BR-3 cell lines were separated in the following cell subpopulations: general (total of cells), positive (ALDH1+ cells) and negative (ALDH1− cells). The subpopulations were grown in mammospheres in suspension (3D culture) and embedded in Matrigel (3D+lrECM culture) during five days, and were irradiated at different doses (0, 2 and 6 Gy). A total of 24 h post-IR, the qPCR was used to measure the expression of the selected MMPs, HDACs and TIMPs. The expression of the genes detected for each cell line, in the different cell subpopulations and for each type of culture, are shown in Supplementary Tables S1 and S2.

**Figure 2:**
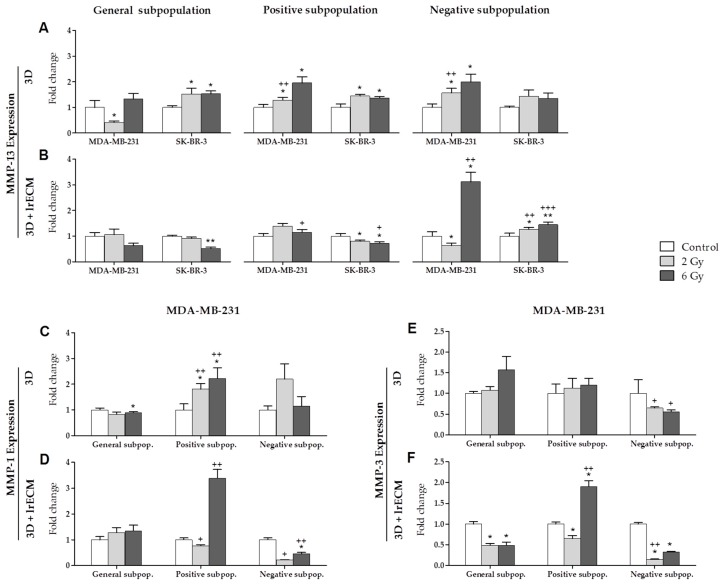
Expression (fold change) of MMP-13 (**A**,**B**), MMP-1 (**C**,**D**) and MMP-3 (**E**,**F**) at 0, 2 and 6 Gy IR doses in the general, positive and negative cell subpopulations of the MDA-MB-231 and SK-BR-3 cell lines in 3D and 3D+lrECM culture models. Values are expressed as median ± SEM (error bars) of three independent experiments run in triplicate. Mean value for each experiment was calculated by averaging the triplicates (*n* = 3). Significant values are marked with * (comparison of IR doses with non-irradiated control) and with + (comparison of positive and negative subpopulations with the general subpopulation.); */+ *p* < 0.05, **/++ *p* < 0.01 and ***/+++ *p* < 0.001.

MMP-13 (Figure 2A,B) was expressed by the MDA-MB-231 and SK-BR-3 lines. In the 3D culture (Figure 2A), the expression of this gene increased with the increase in IR in all cell subpopulations from both cell lines. However, it is worth noting the significant decrease in MMP-13 expression at 2 Gy in the general subpopulation for the MDA-MB-231 line. Besides, MMP-13 expression was also significant in the positive and negative subpopulations to be compared with the general subpopulation at 2 Gy in the same cell line. In the 3D+lrECM culture (Figure 2B), MMP-13 expression tends to decrease with IR in both cell lines except in the negative subpopulation, where it shows a significant increase. This significance was not only found when comparing the different doses within the negative subpopulation, but also when comparing to the general subpopulation. MMP-1 and MMP-3 were expressed by the triple negative MDA-MB-231 cell line (Figure 2C–F). In the 3D culture, MMP-1 expression (Figure 2C,D) showed a significant increase in the positive subpopulation (Figure 2C) for 2 and 6 Gy, and when compared to the general subpopulation at these IR doses. On the other hand, in the 3D+lrECM culture (Figure 2D), MMP-1 expression increased significantly at 6 Gy in the positive subpopulation when compared to the general subpopulation. The negative subpopulation decreased when compared to the general subpopulation at 2 and 6 Gy. In the 3D culture, MMP-3 expression (Figure 2E,F) showed an increase with IR (Figure 2E) in the general and positive subpopulations and a significant decrease in the negative subpopulation. In the 3D+lrECM culture (Figure 2F), MMP-3 expression decreased significantly with IR in the general and negative subpopulations, but in the positive subpopulation it increased significatively at 6 Gy when compared to both the IR doses and the subpopulations.

**Figure 3:**
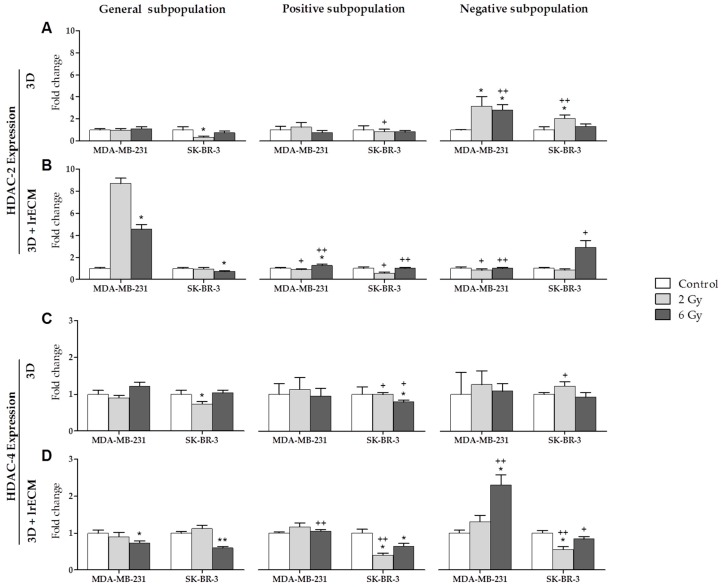
Expression (fold change) of HDAC-2 (**A**,**B**) and HDAC-4 (**C**,**D**) at 0, 2 and 6 Gy IR doses in the general, positive and negative cell subpopulations of the MDA-MB-231 and SK-BR-3 cell lines in 3D and 3D+lrECM culture models. Values are expressed as median ± SEM (error bars) of three independent experiments carried run triplicate. Mean value for each experiment was calculated by averaging the triplicates (*n* = 3). Significant values are marked with * (comparison of IR doses with non-irradiated control) and with + (comparison of positive and negative subpopulations with the general subpopulation.); */+ *p* < 0.05 and **/++ *p* < 0.01.

HDAC-2 was expressed by the MDA-MB-231 and SKBR-3 lines (Figure 3A,B). In the 3D culture (Figure 3A), the expression was about the same in the general and positive subpopulations for both cell lines. In the negative subpopulation, the expression increased significantly for MDA-MB-231 at 2 and 6 Gy and for SK-BR-3 at 2 Gy. These increases were also significant when compared to the general subpopulation at 6 Gy for MDA-MB-231 and at 2 Gy for SK-BR-3. In the 3D+lrECM culture (Figure 3B), HDAC-2 expression was the highest in the general subpopulation for MDA-MB-231, and in the negative subpopulation for SK-BR-3. In both cell lines, the values at 6 Gy were significant with respect to the control. In addition, the expression of this gene was significant when comparing the subpopulations in both cell lines in all cases, except at 2 Gy in the negative subpopulation for SK-BR-3. HDAC-4 was also expressed by the MDA-MB-231 and SK-BR-3 lines (Figure 3C,D). The expression was quite similar in both culture models except for MDA-MB-231 at 6 Gy in the 3D+lrECM negative subpopulation. In the 3D culture (Figure 3C), HDAC-4 expression was significant only for SK-BR-3 when comparing the 2 and 6 Gy doses with the control, and when comparing the positive and negative subpopulations with the general subpopulation. In the 3D+lrECM culture (Figure 3D), both cell lines showed significant values when comparing both the doses and the subpopulations, with the highest increase exhibited by the MDA-MB-231 line at 6 Gy in the negative subpopulation.

**Figure 4:**
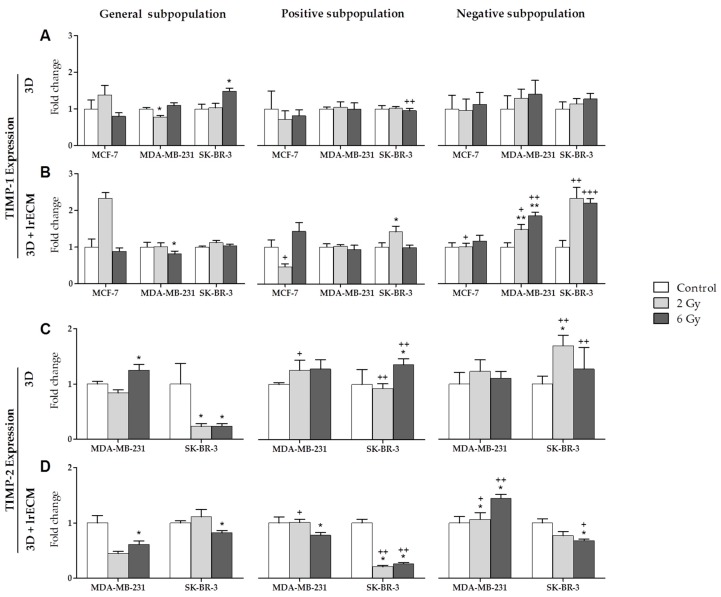
Expression (fold change) of TIMP-1 (**A**,**B**) and TIMP-2 (**C**,**D**) at 0, 2 and 6 Gy in the general, positive and negative cell subpopulations of the MCF-7, MDA-MB-231 and SK-BR-3 cell lines in 3D and 3D+lrECM culture models. Values are expressed as median ± SEM (error bars) of three independent experiments run in triplicate. Mean value for each experiment was calculated by averaging the triplicates (*n* = 3). Significant values are marked with * (comparison of IR doses with non-irradiated control) and with + (comparison of positive and negative subpopulations with the general subpopulation.); */+ *p* < 0.05, **/++ *p* < 0.01 and ***/+++ *p* < 0.001.

Finally, TIMP-1 (Figure 4A,B) was the only one gene expressed by the three cell lines (MCF-7, MDA-MB-231 and SK-BR-3). In the 3D culture (Figure 4A), the expression of this gene was very similar in all subpopulations and was of little significance. In the 3DlrECM culture (Figure 4B), the expression of TIMP-1 increased in the negative subpopulation in the MDA-MB-231 and SK-BR-3 lines. This increase was significant between subpopulations for both cell lines, and between doses only for MDA-MB-231. TIMP-2 (Figure 4C,D) was expressed by the MDA-MB-231 and SK-BR-3 lines. In the 3D culture (Figure 4C), TIMP-2 showed almost no expression changes for the MDA-MB-231 line. However, the expression decreased greatly with IR in the general subpopulation for the SK-BR-3 line. Expression in the positive and negative subpopulations was significant when comparing with the general subpopulation. However, in the 3D+lrECM culture (Figure 4D), TIMP-2 expression was significantly lower in the positive subpopulation for SK-BR-3 than in the general subpopulation. For MDA-MB-231, TIMP-2 expression was significantly higher in the negative subpopulation than in the general subpopulation.

### 2.3. IR Effects on In Vivo Orthotopic Assay

#### 2.3.1. Tumor Growth Monitoring

**Figure 5:**
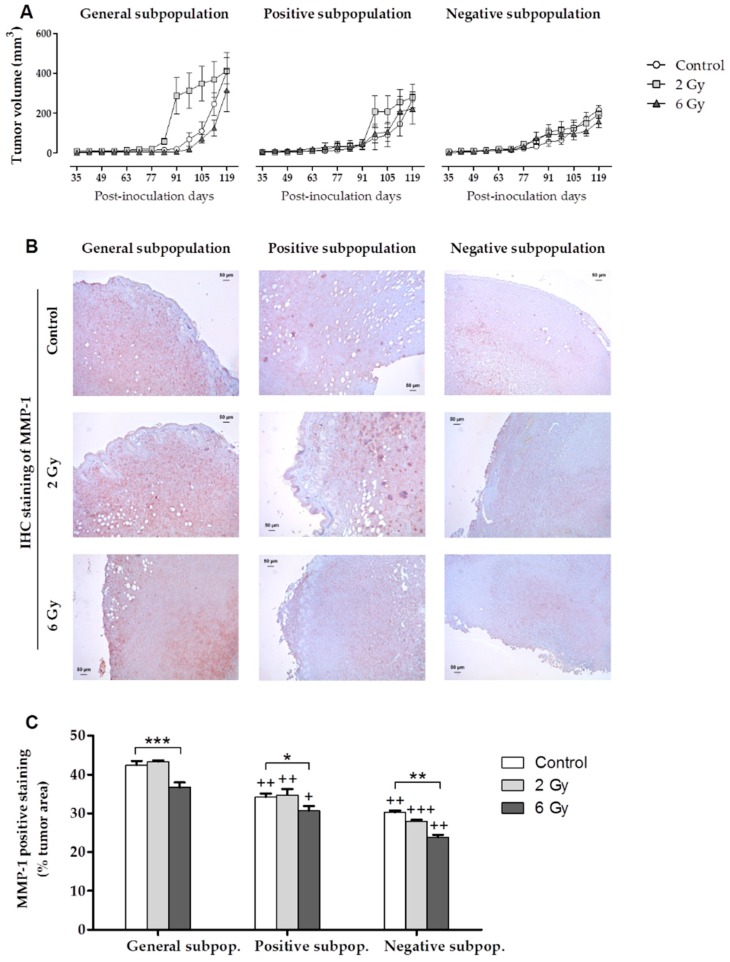
Results of in vivo orthotopic xenotransplant assays. (**A**) Monitoring of tumor growth after orthotopic inoculation in Matrigel of the general, positive and negative cell subpopulations of the MDA-MB-231 cell line at 0, 2 and 6 Gy. Values are expressed as median ± SEM (error bars). Values of tumor volume and *p* values are shown in Supplementary Tables S4–S6. (**B**) IHC staining of MMP-1 in the tumors from the different conditions studied (IR doses, cell subpopulations) for the MDA_MB-231 cell line. (**C**) MMP-1 positive staining (% of the positive area in relation to total tumor area) corresponding to IHC staining of the tumors from the different conditions studied (IR doses and cell subpopulations). Values are expressed as median ± SEM (error bars). Significant values are marked with * (comparison of IR doses with non-irradiated control) and with + (comparison of positive and negative subpopulations with the general subpopulation.); */+ *p* < 0.05, **/++ *p* < 0.01 and ***/+++ *p* < 0.001.

Irradiated and non-irradiated cells from the general, positive and negative subpopulations of the MDA-MB-231 line were injected in the mammary gland of female NSG mice. Figure 5A shows the monitoring of tumor growth performed once a week, until the animals were euthanized 120 days after inoculation. The graph shows the monitoring starting at day 35 in order to better visualize the tumor behavior as tumor size during the first five weeks was very small. Tumor cells were first visible in the positive subpopulation and last in the negative subpopulation. In the control cells from the three subpopulations, the largest tumor was observed in the general subpopulation, and the smallest in the negative subpopulation. In the general subpopulation, tumor size increased at 2 Gy and decreased at 6 Gy IR dose, in both subpopulations when compared to the control. In the positive subpopulation, tumor size followed the same tendency as the general subpopulation, but with lower values. In the negative subpopulation, tumor volume for all IR doses was smaller than in the general and positive subpopulations.

#### 2.3.2. Histological and Immunohistochemical (IHC) Staining

Tumors removed from the mice were fixed in PFA, dipped in paraffin and cut in sections. Sections were stained in Hematoxylin–Eosin (H&E) (Supplementary Figure S1). Based on the color, different cell densities were determined according to the cell subpopulation studied and the dose of irradiation. Cell density was highest in the general subpopulation and smallest in the negative subpopulation.

An IHC staining of MMP-1 (Figure 5B) was also performed and the positive staining percentage of MMP-1 per tumor area (Figure 5C) was calculated. MMP-1 was chosen because it is directly related to the processes of invasion and metastasis and, in the in vitro experiments, this gene was expressed in the two culture models only in the MDA-MB-231 cell line. The highest staining percentage was found in the general subpopulation tumors and lowest in the negative subpopulation tumors. In all subpopulations, staining percentage decreased significantly at 6 Gy. However, in the general and positive subpopulations at the 2 Gy dose, the MMP-1 positive staining percentage increased with respect to the control. This increase was not statistically significant. Finally, significant values were found when comparing the positive and negative subpopulations with the general subpopulation.

## 3. Discussion

We have investigated the influence of IR in the expression of MMPs and their inhibitors in 3D and 3D+lrECM cultures of cell subpopulations from human BC cell lines with different molecular profiles (MCF-7, MDA-MB-231 and SK-BR-3). The present work shows the feasibility of developing an in vitro 3D+lrECM microenvironment that is closer to the in vivo environment. The expression levels of the BCSC markers ALDH1, CD44^+^ and CD24^−/low^ was also evaluated in 2D and 3D cultures after IR, with variations in the expression found between the two culture models used. In general terms, the expression level of ALDH1 decreased with IR dose in the cell lines in both culture models. In contrast, the expression of CD44^+^ increased with IR dose in the three cell lines studied and the selection of cells with a more stemness phenotype. This may be due to cell death of non-CSCs after IR. CD44^+^ has been reported to have the potential to be a marker of poor prognosis and to be involved in radio and chemotherapy resistance in several cancers [28,29,30]. Some authors have demonstrated that cells from tumorigenic cell lines derived from BC and seeded into a 3D Matrigel model, initially undergo cell multiplication without translocating through the gel, forming highly structured spheres. These authors have suggested the possibility that these behaviors were related to tumorigenesis in vivo [31].

Most cancer therapies focus on cancer cells but fail to consider the role of the tumor microenvironment in the regulation of tumor growth and metastasis [32,33]. The ECM and the components of the tumor microenvironment play a crucial role in the regulation of tumor cell function and disease progression [34]. These non-neoplastic components of the tumor microenvironment facilitate tumor growth through the production of ECM, cytokines, growth factors, mechanical cues and vascular networks for nutrient and waste exchange [33]. Therefore, understanding of the role of the tumor microenvironment in intrinsic and acquired radioresistance of tumors is paramount.

The microenvironment also plays a crucial part in maintaining normal stem cells in a quiescent state while preserving their potential for proliferation and differentiation. Thus, identification of the biological processes utilized by CSCs to interact with the surrounding microenvironment will yield crucial information on the role of CSCs in treatment failure and tumor recurrence [35]. Conventional RT targets the bulk of the tumor burden but fails to prevent proliferation and maintenance of CSCs within the tumor [36]. CSCs, which exhibit intrinsic radioresistance, can survive conventional treatment regimens and repopulate the tumor [37] contributing to tumor recurrence and metastasis. CSCs represent the critical population for predicting treatment outcome, and their number and radiosensitivity are important for tumor control after RT. The number of CSCs in tumors has been reported to vary widely, and animal models have shown that the number of (putative) CSCs correlates with the single radiation dose required for tumor control [38].

Studying the triple negative BC MDA-MB-231 cell line, our results show an increase in MMP-1 and MMP-3 expression levels with IR. This increase was markedly higher in cells cultured in 3D+lrECM. We suggest that this culture model could contribute to the appearance of a more aggressive MDA-MB-231 phenotype secondary to IR.

MMP-1 is highly expressed by BC tissues [39] and some authors have found that it may have an effect on the tumor microenvironment [40]. High MMP-1 expression has also been related to a lower overall survival rate in invasive BC [41] and poorer prognosis in patients treated with systemic therapy [42]. Recently, some authors have shown the role of the Y-box binding protein-1 (YB-1) in MDA-MB-231 cell invasion through the regulation of β-catenin and MMP-1 expression, as well as MMP-1 enzymatic activity [43].

MMP-3 has been involved in EMT [44,45], but the relationship between radiation-induced EMT processes and CSCs has not been established conclusively. Though many studies have reported an association between EMT and the gain of CSC properties, the signaling pathways that link them are not clear and could be triggered by TGFβ, Wnt/β-catenin, Hedgehog, Notch, and others [46]. Similarly, IR-induced enrichment of CSCs in xenografts exposed to radiation [47] as well as induction of stem cell-like properties in non-stem cancer cells have also been reported [48,49,50]. CD44 has been reported to be associated with EMT in BC [51]. It has also been proposed that some CD44 isoforms are involved in metastasis and resistance to reactive oxygen species (ROS) and chemotherapy, which is related to poor prognosis [52,53,54,55]. In this respect, Tsubouchi et al. have investigated the role of CD44 isoforms in irradiated pancreatic cancer cells, especially with respect to radioresistance [56]. The same authors have demonstrated that CD44, the standard isoform, was specially upregulated after high-dose X-ray IR contributing to long-term cell survival after the IR through the maintenance of Erk phosphorylation and radiation-induced EMT-like changes in protein expressions.

In general, an increase in MMP-13 expression was related to increasing IR in MDA-MB-231 and SK-BR-3 cell lines grown in the 3D culture for the three subpopulations studied. MMP-13 is an essential protein involved in tumor invasion and metastasis and its expression may help predict the response to RT and prognosis in patients with oral squamous cell carcinoma [57]. It has also been suggested that the highly invasive potential of CSCs depends on MMP-13 enzymatic activity in glioblastoma [58].

HDACs have proved promising targets for anti-cancer drug therapy in preclinical and clinical studies [59,60]. Most HDACs are overexpressed in BC, which leads to the deacetylation of histones and increased cell proliferation, angiogenesis and invasion [61]. We have found that IR induces some alterations in HDAC-2 and HDAC-4, related to the dose administered and subpopulation studied. Some authors have shown the importance of the interaction between HDAC-4 and 53BP1 to mediate the response to DNA damage [62] and stemness behavior and have suggested that HDAC-4 is a strategic component of the complex molecular machinery responsible for radioresistance in some aggressive tumors [63]. HDAC-2 is highly expressed in the more aggressive subgroups of BC [64] and it is a key factor regulating CSC phenotype and in vivo cancer growth in osteosarcoma [65]. Our results showed that HDAC-2 was significantly upregulated with increasing IR dose in the MDA-MB-231 positive subpopulation grown in 3D+lrECM culture, which could be related to the recapitulation of a stem cell niche.

Cid et al. have studied the involvement of MMPs and TIMPs in BC aggressiveness in order to improve the prognostic evaluation of all BC subtypes [66]. Our results show a significant increase in TIMP-1 expression between subpopulations for MDA-MB-321 and SK-BR-3. Moreover, IR significantly increases TIMP-1 expression in MDA-MB-231. Upregulation of TIMP-1 and TIMP-2 led to inhibition of cell migration and invasion in hepatocellular carcinoma cells [67]. It has been shown that overexpression of TIMP-1 has a radiosensitizing effect in a renal carcinoma cell line [68].

We have also investigated the effect of IR on tumor growth through orthotopic inoculation of irradiated MDA-MB-231 cells. Our results show the influence of the different subpopulation interactions on tumor proliferation after IR. Thus, the largest tumor volume was seen in tumor cells from the general subpopulation, with CSCs and non-CSCs. This indicates the relevance of the interaction between CSCs and non-CSCs. The dynamic model of CSCs, which postulates that any differentiated tumor cell can be converted to a CSC, could explain this large tumor volume [8]. In other words, the general subpopulation might have a greater amount of CSCs due to the possible conversion of non-CSCs to CSCs [69]. In our previous studies [70], non-stem ALDH1 negative subpopulations (S-) acquired a CSC-like phenotype after culture in a conditioned medium obtained from secretome of MSCs, which has the ability to select the most aggressive CSC subpopulation. In agreement with our results, evidence has been provided that dedifferentiation of non-tumorigenic tumor cells into CSCs can occur [7]. This conversion is supported by several factors, such as microenvironmental changes, and involves the reactivation of one or more pluripotency genes [71]. CSC phenotype is not a rigid state and not all the CSCs express the same markers. The heterogeneity of cancer also extends to CSCs characteristics. There are different types of CSCs with distinct expression of different biomarkers. For example, no proliferative and quiescent CSCs (CD44^+^/Fbw7^+^/c-Myc^−^) display a different combination of biomarkers compared to proliferative CSCs (CD44^+^/Fbw7^−^/c-Myc^+^) [72]. In a similar way, CSCs with an epithelial phenotype that exhibit the EpCAM biomarker and CSCs with a mesenchymal phenotype that display the CD44 and CD90 biomarkers can found [73]. Moreover, it has been reported that there is a great heterogeneity of CSC-associated biomarkers in breast cancer. For instance, CSCs expressing CD44 and ALDH1 are strongly correlated with a high tumorigenic and chemoresistant phenotype, whereas CSCs expressing CD133 are associated with a high metastatic phenotype in breast cancer [74].

Different mechanisms have been reported to be involved in cell death after irradiation, including apoptosis, autophagy, necrosis, premature senescence and mitotic catastrophe. The activation of a given mechanism is related to the studied cell type and the absorbed radiation dose. Of these, apoptosis and premature senescence have been induced in irradiated MDA-MB-231 cells [75]. As for apoptotic death after irradiation, our results confirm the radioresistance of the MDA-MB-231 CSC positive subpopulation (Figure S2 and Table S8). In addition, these results support the inability of a dose of 2 Gy to eradicate the CSC subpopulation and the capacity of this dose to even promote tumor growth. This effect was more pronounced in tumors from the general subpopulation, which suggests the importance of CSC and non-CSC interactions in tumor proliferation after IR. These data also support the hypothesis that non-CSCs exhibit a remarkable degree of plasticity that allows them to re-acquire CSC traits, especially in the context of RT [48]. CSCs with properties of radioresistance are speculated to be responsible for the repopulation of cancer cells after RT, contributing to disease relapse. In contrast, higher IR doses (6 Gy) are associated with tumor growth delay, probably due to CSC apoptotic death (Figure S2 and Table S8). Nevertheless, these results have been obtained in a xenograft model which clearly requires immunodeficient mice. This lack of a functional immune system in the host mice is a limitation for clinical translation.

It has been suggested that CSC survival can be impacted by their niches following the stress of IR exposure [76,77,78,79]. The niche-associated Notch pathway was shown to be activated after IR and resulted in increased symmetric cell division and accelerated repopulation of CSCs [80]. Notch activation has also been suggested to activate the EGFR pathway, which could promote DNA repair capability, CSC survival and regeneration kinetics [81]. Moreover, CSC niches may also produce survival cytokines, such as EGF, FGF, and VEGF, all of which are responsible for the radioresistance and radioprotection of cancer cells [76].

Our results suggest a differential expression of matrix enzymes and their inhibitors related to the use of the single radiation dose administered, the molecular subtype assayed and the type of cultures model used. They indicate that the selection of IR doses depending on niche and tumor characteristics is essential to have efficacy in eliminating resistant CSCs subpopulations in BC. Some authors have demonstrated specific molecular patterns, including increased activity of metalloproteinases characteristic for BC and metastatic disease, with particularly poor outcomes [82]. In this sense, genotyping is expected to complete the individual portrait of triple-negative BC particularly predisposed to aggressive metastatic disease [83]. It would be of great interest to establish a CSC molecular signature in BC by studying changes in gene and protein expression in response to radiation in cellular, animal model systems and patients. In view of these results, further studies are needed to understand the pathways and specific mechanisms of radioresistance and how to sensitize and target CSCs with RT to have success in the treatment of BC patients.

## 4. Materials and Methods

### 4.1. Cell Lines

Human BC cell lines, MCF-7, MDA-MB-231 and SK-BR-3, were obtained from the American Type Culture Collection (ATCC) and maintained as a monolayer culture in DMEM medium (Dulbecco’s Modified Eagle’s Medium; HyClone, Waltham, MA, USA) supplemented with 1% Penicillin–Streptomycin Solution 100× (Gibco), 1% Amphotericin B Solution (250 μg/mL) (Sigma-Aldrich, Saint Louis, MO, USA), and 10% Fetal Bovine Serum (FBS) (Lonza, Basel, Switzerland). Cells were cultured at 37 °C in a 5% CO_2_ atmosphere. Cell lines were maintained in these conditions until all experiments were carried out.

### 4.2. Flow Cytometry Analysis

#### 4.2.1. BCSC Characterization

For the characterization of BCSCs, cell lines MCF-7, MDA-MB-231 and SK-BR-3 were grown in 2D culture as monolayer and in 3D culture as mammospheres in suspension (only the positive subpopulation). These mammospheres were obtained by sorting with the Aldefluor kit (see Section 4.3.1.). After five days, cell lines were irradiated at 0, 2 and 6 Gy and characterization was performed 24 h after irradiation. The markers determined were the specific human antibodies anti-CD24 (APC) and anti-CD44 (PE) (Miltenyi Biotec, Bergisch Gladbach, Germany) and the enzyme ALDH1 activity was also detected using the Aldefluor Kit (StemCell Technologies, Vancouver, BC, Canada). Samples were analyzed on a BD FACSCanto II flow cytometer (Becton Dickinson, Franklin Lakes, NJ, USA). Three independent experiments were run in triplicate. The mean value for each experiment was calculated by averaging the triplicates (*n* = 3).

#### 4.2.2. Apoptotic Cell Identification

Apoptosis was measured 24 h after irradiation. Cell death was analyzed by the eBiosciencie™ Annexin V-FITC Apoptosis detection kit (Invitrogen, Carlsbad, CA, USA) and PI staining according to the manufacturer’s instructions. In short, the different cell subpopulations treated with radiation were harvested, washed, and suspended in Annexin V and Binding Buffer (Invitrogen). The Annexin V-FITC (2.5 µg/mL) and PI solution (20 µg/mL) were added to the cell suspension and incubated for 15 min at room temperature in the dark. Then, the apoptotic cells were analyzed using flow cytometry (BD FACSCanto II flow cytometer, Becton Dickinson). Three independent experiments were run in triplicate. The mean value for each experiment was calculated by averaging the triplicates (*n* = 3).

### 4.3. In Vitro Measurements of Gene Expression

#### 4.3.1. Separation of Cell Subpopulations

The Aldefluor kit (StemCell Technologies) was used to separate the subpopulations of MCF-7, MDA-MB-231 and SK-BR-3 cell lines, according to manufacturer’s instructions. The separation was carried out in a BD FACSAria II flow cytometer (Becton Dickinson). The following subpopulations were isolated: CSCs or positive subpopulation (ALDH1+ cells) and non-CSCs or negative subpopulation (ALDH1− cells). The third subpopulation was completed when passing the cell lines without the Aldeflour assay by the cytometer. The third subpopulation was completed when passing the cell lines without the Aldeflour assay by the cytometer. This subpopulation was named the general subpopulation because it contains the total of the cells and it was used as control subpopulation. The three subpopulations from the three cell lines were maintained in 3D and 3D+lrECM culture models during the experiment.

##### 3D Culture (Mammospheres in Suspension)

Cell subpopulations were cultured in suspension on ultra-low attachment plates in spheres medium DMEM-F12 (Dulbecco’s Modified Eagl’s Medium/Nutrient Mixture F-12 Ham; Sigma-Aldrich), supplemented with 1% Penicillin–Streptomycin Solution 100× (Gibco), 1 mg/mL hydrocortisone (Sigma-Aldrich), 4 ng/mL heparin (Sigma-Aldrich), 1× ITS (Gibco), 1× B27 (Gibco), 10 ng/mL EGF (Sigma-Aldrich) and 10 ng/mL FGF (Sigma-Aldrich).

##### 3D+lrECM Culture (3D Laminin-Rich ECM)

Cell subpopulations were cultured on ultra-low attachment plates by embedding the cells in 0.5 mg/mL lrECM (Matrigel; Corning, NY, USA, 20 mg/mL), which was prepared with DMEM-F12 medium. The use of the Matrigel and the preparation of the lrECM were carried out according to manufacturer’s instructions.

#### 4.3.2. Irradiation Protocol

Five days after the separation of the different subpopulations, the cells were irradiated at two different doses: 2 Gy and 6 Gy using an Yxlon Smart Maxishot 200-E at room temperature, 4.5 mA current and 200 kW power. Non-irradiated cells were used as the control. After IR, cells were cultured for 24 h.

#### 4.3.3. cDNA Amplification and Quantitative Real-Time Polymerase Chain Reaction (qRT-PCR)

At 24 h post-IR, total RNA from the different cell subpopulations (non-irradiated and irradiated cells) of MCF-7, MDA-MB-231 and SK-BR-3 cell lines were isolated and purified using an RNeasy kit (Qiagen, Hilden, Germany). An equal amount of RNA from the samples was reverse-transcribed to cDNA and amplified by PCR with the iScript cDNA Synthesis Kit (BioRad, Hercules, CA, USA) according to manufacturer’s instructions. The qRT-PCR assay was performed using the SsoFast EvaGreen Supermix kit (BioRad). Expression of the genes MMP-1, -2, -3, -9, -13, TIMP-1, -2, HDAC-1, -2 and -4 (Sigma-Aldrich) was measured according to the manufacturer’s protocol and the values obtained were normalized with two reference genes, 18S and GAPDH. Primer sequences used are described in Supplementary Table S1. Three independent experiments were run in triplicate. The mean value for each experiment was calculated by averaging the triplicates (*n* = 3).

### 4.4. In Vivo Orthotopic Xenotransplant Assays

#### 4.4.1. Inoculation of Cells in Matrigel and Monitoring of Tumor Growth

Orthotopic assays were carried out in the general, positive and negative cell subpopulation of the MDA-MB-231 cell line. Cell subpopulations were cultured in sphere medium and irradiated at 0, 2 and 6 Gy. At 24 h post-IR, 3000 cells from each subpopulation were injected in a mixture of 0.05 mL Matrigel (Corning, 20 mg/mL) and 0.05 mL DMEM-F12 (Sigma-Aldrich) into the mammary fat pad of 8-week-old NOD scid gamma mice (NOD.Cg-Prkdcscid Il2rgtm1Wjl/SzJ, NSG). Five mice were used per dose of radiation, 15 mice per cell subpopulation, which means a total of 45 mice. A tumor was obtained from each mouse, with a total of 5 tumors per study condition. Tumor growth was evaluated once a week using a digital caliper. Tumor volume was calculated using the equation V = length2 × width × π/6. The mice were euthanized 120 days after injection and the tumors removed for analysis. A tumor was obtained from each mouse, with a total of 5 tumors per study condition. Animal experimentation was performed according to the protocols reviewed and approved by the Institutional Animal Care and Use Committee of the University of Granada (PI730/13).

#### 4.4.2. Histological and Immunohistochemical (IHC) Assays

Tumors were fixed in 4% paraformaldehyde in 0.1 M PBS at 4 °C for 24 h, washed in 0.1 M PBS and subsequently immersed in paraffin in an automatic tissue processor (TP1020, Leica, Wetzlar, Germany). Paraffin blocks were cut into 5 μm sections. For Hematoxylin and Eosin (H&E) staining, the sections were hydrated (de-paraffinized), stained with Hematoxylin and Eosin (Sigma-Aldrich), and dehydrated according to manufacturer’s instructions.

For the IHC assays, the sections were hydrated and incubated with 10 mM citrate buffer for 20 min at 100 °C, washed three times in Tris Buffered Saline (TBS; Tris 50 mM and NaCl 0.15 M) for 5 min and incubated in 3% H_2_O_2_ in methanol for 10 min. Subsequently, the sections were washed with tap water, and IHC analysis of MMP-1 (anti-MMP1; Abcam, Cambridge, UK) was performed using the Vectastain Elite ABC Kit (Vector Laboratories). Lastly, the sections were incubated in Dako Real DAB + Chromogen (Dako) for staining development for 90 s. Hematoxylin was used as counterstain.

Three sections from each tumor were cut and stained. A section from each tumor was chosen and four microphotographs were taken at 10× magnification. Digital micrographs were acquired using a Leica DMI3000B microscope (Barcelona, Spain) equipped with a Leica DFC420 C Camera. The IHC quantification has been done using ImageJ™ software (v1.44.) and data were calculated as the positive staining percentage of MMP-1 per tumor area.

### 4.5. Statistical Analysis

The results were expressed as mean ± standard error of the mean (SEM). The statistical analysis was performed with the IBM SPSS Statistics software package v23.0, while GraphPad Prism 8.0.0 was used for plotting the data sets; *p* values < 0.05 were considered significant. Given the limited samples size and the non-normal distribution of variables, Mann–Whitney U and Kruskal–Wallis non-parametric tests were used for comparison between two or more groups, respectively. Significant differences were indicated differently (* when the IR doses are compared with the non-irradiated control, and + when the positive and negative subpopulations are compared with the general subpopulation); ^*/+^ for *p* < 0.05; ^**/++^ for *p* < 0.01; and ^***/+++^ for *p* < 0.001.

## 5. Conclusions

- The expression of the BCSCs (ALDH1, CD44^+^ and CD24^−/low^) varies with the dose of radiation administered. In the positive subpopulation (CSCs), high doses of radiation (6 Gy) increases CD44^+^, which is associated with EMT and a poor prognosis in BC.
- In the in vitro 3D and 3D+lrECM studies, the expression of MMPs, TIMPs and HDACs varies depending on the tumor cell line (MCF-7, MDA-MB-231 and SK-BR-3), the radiation dose (0, 2 and 6 Gy), the cell subpopulation (general, positive or CSCs, and negative or non-CSCs) and the 3D culture model (spheres suspended or embedded in Matrigel).
- MMP-1 expression was increased in both 3D culture models after radiation in the positive subpopulation (CSCs) of the MDA-MB-231 triple-negative cell line. This fact suggests the importance of MMP-1 in the process of invasion and metastasis.
- In the in vivo study, tumors derived from cells irradiated at 2 Gy have been of higher volume with respect to the control in the general and positive subpopulations. This fact shows that low doses of radiation would be insufficient to eradicate CSCs due to their radioresistance, contributing to repopulation and the tumor’s growth.
- In tumor development in vivo, tumors derived from cells irradiated at 6 Gy were the smallest within each cell subpopulation. This result suggests that high doses of radiation (6 Gy) counteract repopulation by reducing the tumor growth rate and the final volume of the tumor.

## Supplementary Materials

The following are available online at https://www.mdpi.com/2072-6694/11/11/1781/s1, Figure S1: H&E staining of the tumors for all IR doses (0, 2 and 6 Gy) in the general, positive and negative cell subpopulations of the MDA-MB-231 cell line, Figure S2: Values of apoptosis (%) at 0, 2 and 6 Gy IR doses measured 24 h after treatment in MCF-7 (A), MDA-MB-231 (B) and SK-BR-3 (C) cell lines in the general subpopulation and positive subpopulation cultures, Table S1: Expression values (fold change) of the genes detected in the 3D culture for all IR doses (0, 2 and 6 Gy) in the general, positive and negative cell subpopulations of the MCF-7, MDA-MB-231 and SK-BR-3 cell lines, Table S2: Expression values (fold change) of the genes detected in the 3D + lrECM culture for all IR doses (0, 2 and 6 Gy) in the general, positive and negative cell subpopulations of the MCF-7, MDA-MB-231 and SK-BR-3 cell lines, Table S3: Primer sequences used for the measurements of gene expression in qRT-PCR assays, Table S4: Values of tumor volume (mm^3^) corresponding to monitoring of tumor growth after orthotopic inoculation in Matrigel of the general, positive and negative cell subpopulations of the MDA-MB-231 cell line at 0, 2 and 6 Gy, Table S5: *p* values of tumor volume when comparing the doses of 2 and 6 Gy with the control within each cell subpopulation, Table S6: *p* values of tumor volume when comparing the positive and negative subpopulations with the general subpopulations, Table S7: Values of MMP-1 positive staining (% tumor area) corresponding to IHC staining of the tumors from the different conditions studied (IR doses and cell subpopulations), Table S8: Values of apoptosis (%) of the MCF-7 (A), MDA-MB-231 (B) and SK-BR-3 (C) cell lines in the general subpopulation and positive subpopulation cultures at 0, 2 and 6 Gy IR doses.
