# Supplementary material for: Radiation and Stemness Phenotype May Influence Individual Breast Cancer Outcomes: The Crucial Role of MMPs and Microenvironment
Source: Cancers (Basel). 2019 Nov 12;11(11):1781. doi: 10.3390/cancers11111781 (PMC6896076; doi:10.3390/cancers11111781)
Supplement: Supplementary file 1 [file cancers-11-01781-s001.pdf]

*Supplementary Materials*

# Radiation and Stemness Phenotype May Influence Individual Breast Cancer Outcomes: The Crucial Role of MMPs and Microenvironment

**María Auxiliadora Olivares-Urbano, Carmen Griñán-Lisón, Sandra Ríos-Arrabal, Francisco Artacho-Cordón, Ana Isabel Torralbo, Elena López-Ruiz, Juan Antonio Marchal and María Isabel Núñez**

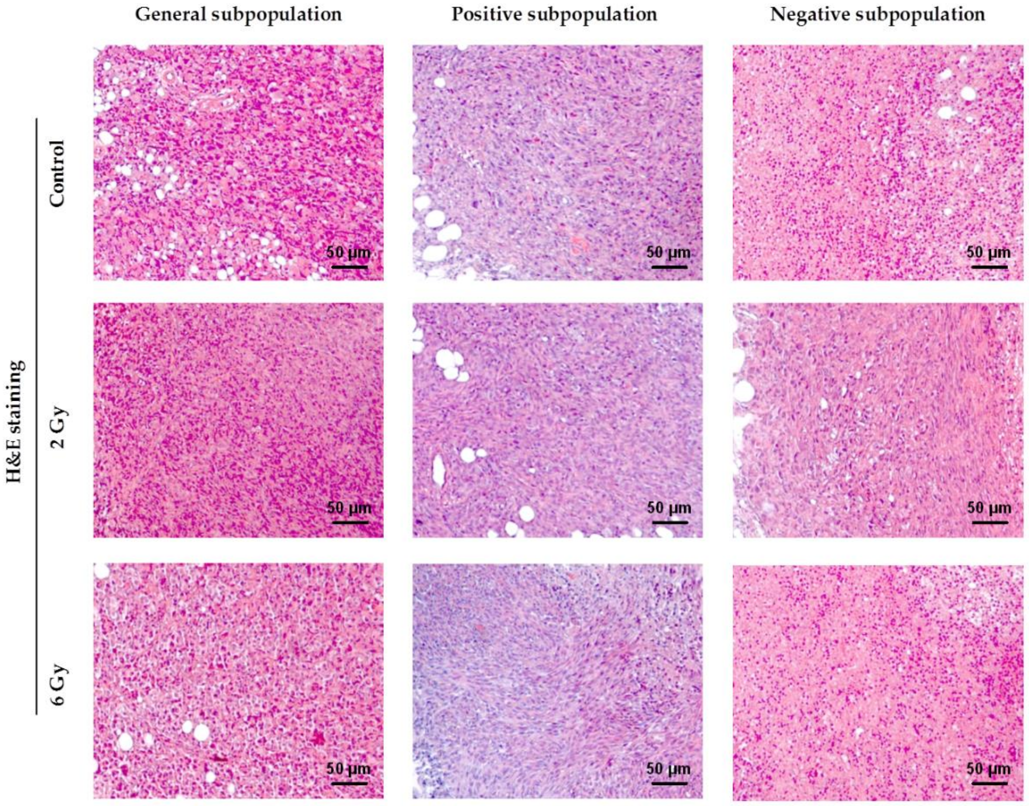


**Figure S1.** H&E staining of the tumors for all IR doses (0, 2 and 6 Gy) in the general, positive and negative cell subpopulations of the MDA-MB-231 cell line.

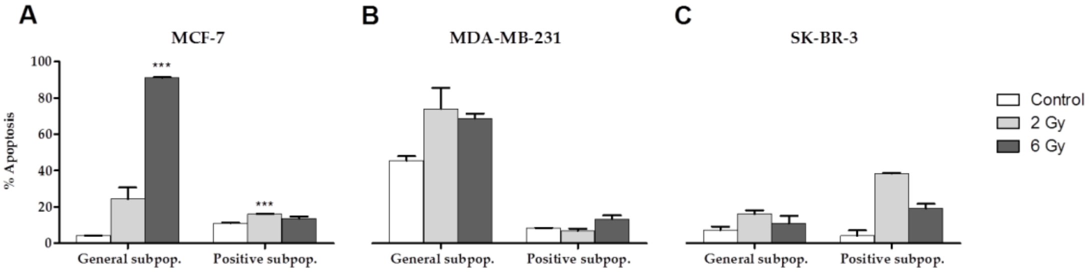


**Figure S2.** Values of apoptosis (%) at 0, 2 and 6 Gy IR doses measured 24 h after treatment in MCF-7 (**A**), MDA-MB-231 (**B**) and SK-BR-3 (**C**) cell lines in the general subpopulation and positive subpopulation cultures. Values are expressed as the mean ± SEM (error bars) of three independent experiments. Mean value for each experiment was calculated by averaging the triplicates ($n = 3$); *** $p < 0.001$.

**Table S1.** Expression values (fold change) of the genes detected in the 3D culture for all IR doses (0, 2 and 6 Gy) in the general, positive and negative cell subpopulations of the MCF-7, MDA-MB-231 and SK-BR-3 cell lines.

| Genes | IR Dose | Expression Values (Fold Change) | | |
|---|---|---|---|---|
| | | General Subpopulation | Positive Subpopulation | Negative Subpopulation |
| **MCF-7** | | | | |
| *TIMP-1* | Control | 1.000 | 1.000 | 1.000 |
| | 2 Gy | 1.378 | 0.719 | 0.961 |
| | 6 Gy | 0.793 | 0.810 | 1.129 |
| **MDA-MB-231** | | | | |
| *MMP-1* | Control | 1.000 | 1.000 | 1.000 |
| | 2 Gy | 0.819 | 1.804 | 2.197 |
| | 6 Gy | 0.898 | 2.224 | 1.146 |
| *MMP-3* | Control | 1.000 | 1.000 | 1.000 |
| | 2 Gy | 1.075 | 1.130 | 0.646 |
| | 6 Gy | 1.567 | 1.202 | 0.556 |
| *MMP-9* | Control | 1.000 | 1.000 | 1.000 |
| | 2 Gy | 0.570 | 2.377 | 0.774 |
| | 6 Gy | 0.655 | 1.584 | 0.675 |
| *MMP-13* | Control | 1.000 | 1.000 | 1.000 |
| | 2 Gy | 0.413 | 1.286 | 1.563 |
| | 6 Gy | 1.326 | 1.960 | 1.991 |
| *HDAC-1* | Control | 1.000 | 1.000 | 1.000 |
| | 2 Gy | 0.740 | 1.234 | 0.441 |
| | 6 Gy | 1.006 | 0.751 | 0.489 |
| *HDAC-2* | Control | 1.000 | 1.000 | 1.000 |
| | 2 Gy | 0.980 | 1.256 | 3.150 |
| | 6 Gy | 1.104 | 0.772 | 2.824 |
| *HDAC-4* | Control | 1.000 | 1.000 | 1.000 |
| | 2 Gy | 0.900 | 1.131 | 1.266 |
| | 6 Gy | 1.219 | 0.950 | 1.089 |
| *TIMP-1* | Control | 1.000 | 1.000 | 1.000 |
| | 2 Gy | 0.777 | 1.048 | 1.290 |
| | 6 Gy | 1.090 | 0.991 | 1.400 |
| *TIMP-2* | Control | 1.000 | 1.000 | 1.000 |
| | 2 Gy | 0.837 | 1.254 | 1.230 |
| | 6 Gy | 1.250 | 1.280 | 1.104 |
| **SK-BR-3** | | | | |
| *MMP-1* | Control | 1.000 | 1.000 | 1.000 |
| | 2 Gy | 0.384 | 0.822 | 1.160 |
| | 6 Gy | 0.764 | 0.538 | 2.398 |
| *MMP-13* | Control | 1.000 | 1.000 | 1.000 |
| | 2 Gy | 1.521 | 1.450 | 1.443 |
| | 6 Gy | 1.537 | 1.367 | 1.351 |
| *HDAC-1* | Control | 1.000 | 1.000 | 1.000 |
| | 2 Gy | 0.578 | 2.944 | 1.097 |
| | 6 Gy | 0.838 | 0.988 | 4.041 |
| *HDAC-2* | Control | 1.000 | 1.000 | 1.000 |
| | 2 Gy | 0.342 | 0.869 | 2.051 |
| | 6 Gy | 0.754 | 0.843 | 1.330 |
| *HDAC-4* | Control | 1.000 | 1.000 | 1.000 |
| | 2 Gy | 0.730 | 1.003 | 1.221 |
| | 6 Gy | 1.035 | 0.792 | 0.924 |
| *TIMP-1* | Control | 1.000 | 1.000 | 1.000 |
| | 2 Gy | 1.030 | 1.017 | 1.141 |
| | 6 Gy | 1.480 | 0.954 | 1.272 |
| *TIMP-2* | Control | 1.000 | 1.000 | 1.000 |
| | 2 Gy | 0.235 | 0.927 | 1.692 |
| | 6 Gy | 0.238 | 1.358 | 1.271 |

**Table S2.** Expression values (fold change) of the genes detected in the 3D + lrECM culture for all IR doses (0, 2 and 6 Gy) in the general, positive and negative cell subpopulations of the MCF-7, MDA-MB-231 and SK-BR-3 cell lines.

| Genes | IR Dose | Expression Values (Fold Change) | | |
|---|---|---|---|---|
| | | General Subpopulation | Positive Subpopulation | Negative Subpopulation |
| **MCF-7** | | | | |
| | Control | 1.000 | 1.000 | 1.000 |
| ***TIMP-1*** | 2 Gy | 2.331 | 0.468 | 1.006 |
| | 6 Gy | 0.886 | 1.432 | 1.167 |
| **MDA-MB-231** | | | | |
| | Control | 1.000 | 1.000 | 1.000 |
| ***MMP-1*** | 2 Gy | 1.276 | 0.771 | 0.224 |
| | 6 Gy | 1.346 | 3.385 | 0.455 |
| | Control | 1.000 | 1.000 | 1.000 |
| ***MMP-3*** | 2 Gy | 0.485 | 0.653 | 0.148 |
| | 6 Gy | 0.482 | 1.906 | 0.326 |
| | Control | 1.000 | 1.000 | 1.000 |
| ***MMP-13*** | 2 Gy | 1.058 | 1.383 | 0.631 |
| | 6 Gy | 0.632 | 1.149 | 3.123 |
| | Control | 1.000 | 1.000 | 1.000 |
| ***HDAC-2*** | 2 Gy | 8.690 | 0.881 | 0.845 |
| | 6 Gy | 4.539 | 1.265 | 1.007 |
| | Control | 1.000 | 1.000 | 1.000 |
| ***HDAC-4*** | 2 Gy | 0.903 | 1.171 | 1.311 |
| | 6 Gy | 0.733 | 1.052 | 2.307 |
| | Control | 1.000 | 1.000 | 1.000 |
| ***TIMP-1*** | 2 Gy | 1.015 | 1.027 | 1.478 |
| | 6 Gy | 0.818 | 0.933 | 1.850 |
| | Control | 1.000 | 1.000 | 1.000 |
| ***TIMP-2*** | 2 Gy | 0.448 | 1.009 | 1.066 |
| | 6 Gy | 0.615 | 0.777 | 1.446 |
| **SK-BR-3** | | | | |
| | Control | 1.000 | 1.000 | 1.000 |
| ***MMP-13*** | 2 Gy | 0.908 | 0.799 | 1.257 |
| | 6 Gy | 0.523 | 0.720 | 1.446 |
| | Control | 1.000 | 1.000 | 1.000 |
| ***HDAC-2*** | 2 Gy | 0.961 | 0.567 | 0.852 |
| | 6 Gy | 0.722 | 1.005 | 2.920 |
| | Control | 1.000 | 1.000 | 1.000 |
| ***HDAC-4*** | 2 Gy | 1.120 | 0.399 | 0.558 |
| | 6 Gy | 0.598 | 0.640 | 0.840 |
| | Control | 1.000 | 1.000 | 1.000 |
| ***TIMP-1*** | 2 Gy | 1.121 | 1.421 | 2.329 |
| | 6 Gy | 1.034 | 0.984 | 2.203 |
| | Control | 1.000 | 1.000 | 1.000 |
| ***TIMP-2*** | 2 Gy | 1.111 | 0.208 | 0.772 |
| | 6 Gy | 0.823 | 0.264 | 0.680 |

**Table S3.** Primer sequences used for the measurements of gene expression in qRT-PCR assays.

| Genes | Primer Sequences | |
|---|---|---|
| *18S* | Forward | CGGCGACGACCCATTCGAAC |
| | Reverse | GAATCGAACCCTGATTCCCCG |
| *GADPH* | Forward | CACCAGGGCTGCTTTTAACTC |
| | Reverse | CCTTGACGGTGCCATGGAATT |
| *MMP-1* | Forward | AAGGCCAGTATGCACAGCTT |
| | Reverse | TGCTTGACCCTCAGAGACCT |
| *MMP-2* | Forward | TTTCCATTCCGCTTCCAGGGC |
| | Reverse | TCGCACACCACATCTTTCCGT |
| *MMP-3* | Forward | GGCTTTCCCAAGCAAATAGC |
| | Reverse | GTGCCCATATTGTGCCTTCT |
| *MMP-9* | Forward | CCTGCCAGTTTCCATTCATC |
| | Reverse | GCCATTCACGTCGTCCTTAT |
| *MMP-13* | Forward | AACATCCAAAAACGCCAGAC |
| | Reverse | GGAAGTTCTGGCCAAAATGA |
| *TIMP-1* | Forward | ACCAGACCACCTTATACCA |
| | Reverse | CATTCCTCACAGCCAACA |
| *TIMP-2* | Forward | TGCTTCTTGCCTGTTCTG |
| | Reverse | CTGGACTTCTTACTATGCTTA |
| *HDAC-1* | Forward | AAGACGACCCTGACAAGC |
| | Reverse | AGACCTGGCACCCTTTAT |
| *HDAC-2* | Forward | GGTAACTCCTGCTACTAAGAT |
| | Reverse | GGTGCTATTGTGAATGTCTG |
| *HDAC-4* | Forward | ACCTACCTGATGCCTGTT |
| | Reverse | AACCTCCAACTCCACTGA |

**Table S4.** Values of tumor volume (mm$^3$) corresponding to monitoring of tumor growth after orthotopic inoculation in Matrigel of the general, positive and negative cell subpopulations of the MDA-MB-231 cell line at 0, 2 and 6 Gy.

| Days | IR Dose | Tumor Volume (mm$^3$) | | |
|---|---|---|---|---|
| | | General Subpopulation | Positive Subpopulation | Negative Subpopulation |
| **7** | Control | 0.000 | 0.000 | 0.000 |
| | 2 Gy | 0.000 | 0.000 | 0.000 |
| | 6 Gy | 0.000 | 0.000000 | 0.000 |
| **14** | Control | 0.000 | 0.000 | 0.000 |
| | 2 Gy | 2.339 | 0.936 | 0.000 |
| | 6 Gy | 0.000 | 1.872 | 0.000 |
| **21** | Control | 0.000 | 2.495 | 0.000 |
| | 2 Gy | 7.798 | 1.872 | 0.000 |
| | 6 Gy | 0.000 | 2.495 | 0.000 |
| **28** | Control | 7.486 | 6.238 | 8.110 |
| | 2 Gy | 10.92 | 2.495 | 3.119 |
| | 6 Gy | 0.000 | 4.387 | 1.248 |
| **35** | Control | 7.486 | 6.238 | 8.110 |
| | 2 Gy | 10.92 | 5.010 | 6.238 |
| | 6 Gy | 2.495 | 8.842 | 2.495 |
| **42** | Control | 7.486 | 6.238 | 8.110 |
| | 2 Gy | 10.92 | 5.010 | 11.22 |
| | 6 Gy | 4.991 | 10.89 | 6.238 |
| **49** | Control | 9.982 | 9.982 | 8.110 |
| | 2 Gy | 10.92 | 5.010 | 12.48 |
| | 6 Gy | 4.991 | 11.02 | 7.486 |

| | | | | |
|---|---|---|---|---|
| **56** | Control | 9.982 | 9.982 | 11.85 |
| | 2 Gy | 10.92 | 7.468 | 14.41 |
| | 6 Gy | 4.991 | 17.70 | 10.00 |
| **63** | Control | 9.982 | 10.63 | 13.76 |
| | 2 Gy | 14.14 | 11.49 | 20.20 |
| | 6 Gy | 4.991 | 20.73 | 14.16 |
| **70** | Control | 13.76 | 10.92 | 15.05 |
| | 2 Gy | 18.96 | 16.36 | 20.20 |
| | 6 Gy | 4.99 | 30.36 | 19.32 |
| **77** | Control | 15.05 | 14.11 | 22.77 |
| | 2 Gy | 20.57 | 35.05 | 45.88 |
| | 6 Gy | 4.991 | 30.36 | 32.080 |
| **84** | Control | 17.63 | 22.86 | 33.44 |
| | 2 Gy | 58.77 | 35.95 | 58.92 |
| | 6 Gy | 4.991 | 31.33 | 75.79 |
| **91** | Control | 20.81 | 40.88 | 57.40 |
| | 2 Gy | 287.3 | 43.89 | 104.2 |
| | 6 Gy | 7.486 | 44.308 | 93.70 |
| **98** | Control | 69.46 | 76.85 | 63.91 |
| | 2 Gy | 313.3 | 208.0 | 118.0 |
| | 6 Gy | 19.44 | 97.76 | 96.27 |
| **105** | Control | 110.1 | 90.14 | 118.3 |
| | 2 Gy | 348.7 | 208.0 | 126.6 |
| | 6 Gy | 72.96 | 106.9 | 96.27 |
| **112** | Control | 245.66 | 146.9 | 174.0 |
| | 2 Gy | 368.96 | 256.1 | 149.8 |
| | 6 Gy | 126.1 | 209.5 | 110.9 |
| **119** | Control | 408.1 | 268.4 | 217.5 |
| | 2 Gy | 413.2 | 279.0 | 194.4 |
| | 6 Gy | 314.0 | 221.3 | 159.2 |

**Table S5.** *p* values of tumor volume when comparing the doses of 2 and 6 Gy with the control within each cell subpopulation.

| **Days** | ***p* Values** | | | | | |
|---|---|---|---|---|---|---|
| | **General Subpopulation** | | **Positive Subpopulation** | | **Negative Subpopulation** | |
| | **2 Gy vs. Control** | **6 Gy vs. Control** | **2 Gy vs. Control** | **6 Gy vs. Control** | **2 Gy vs. Control** | **6 Gy vs. Control** |
| **7** | 1.000 | 1.000 | 1.000 | 1.000 | 1.000 | 1.000 |
| **14** | 0.180 | 1.000 | 0.317 | 0.317 | 1.000 | 1.000 |
| **21** | 0.059 | 1.000 | 0.655 | 1.000 | 1.000 | 1.000 |
| **28** | 0.414 | 0.063 | 0.180 | 0.715 | 0.038 | 0.042 |
| **35** | 0.414 | 0.194 | 0.655 | 0.500 | 0.180 | 0.066 |
| **42** | 0.414 | 0.581 | 0.655 | 0.593 | 0.180 | 0.180 |
| **49** | 0.655 | 0.317 | 0.141 | 1.000 | 0.102 | 0.317 |
| **56** | 0.655 | 0.317 | 0.500 | 0.593 | 0.109 | 0.180 |
| **63** | 0.141 | 0.317 | 0.892 | 1.000 | 0.102 | 0.317 |
| **70** | 0.276 | 0.102 | 0.892 | 1.000 | 0.102 | 0.593 |
| **77** | 0.269 | 0.102 | 0.892 | 0.686 | 0.144 | 0.686 |
| **84** | 0.144 | 0.102 | 1.000 | 0.500 | 0.225 | 0.225 |
| **91** | 0.068 | 0.068 | 0.715 | 0.500 | 0.225 | 0.225 |
| **98** | 0.144 | 0.138 | 0.080 | 0.500 | 0.225 | 0.345 |
| **105** | 0.144 | 0.500 | 0.080 | 0.500 | 0.893 | 0.686 |
| **112** | 0.465 | 0.043 | 0.043 | 0.500 | 0.686 | 0.225 |
| **119** | 0.715 | 0.080 | 0.893 | 0.500 | 0.686 | 0.225 |

**Table S6.** *p* values of tumor volume when comparing the positive and negative subpopulations with the general subpopulations.

| Days | IR Dose | *p* Values | |
|---|---|---|---|
| | | **Positive Subpop. vs. General Subpop.** | **Negative Subpop. vs. General Subpop.** |
| **7** | Control | 1.000 | 1.000 |
| | 2 Gy | 1.000 | 1.000 |
| | 6 Gy | 1.000 | 1.000 |
| **14** | Control | 1.000 | 1.000 |
| | 2 Gy | 0.383 | 0.094 |
| | 6 Gy | 0.317 | 1.000 |
| **21** | Control | 0.136 | 1.000 |
| | 2 Gy | 0.068 | 0.006 |
| | 6 Gy | 0.136 | 1.000 |
| **28** | Control | 0.746 | 0.911 |
| | 2 Gy | 0.022 | 0.022 |
| | 6 Gy | 0.136 | 0.134 |
| **35** | Control | 0.746 | 0.911 |
| | 2 Gy | 0.205 | 0.223 |
| | 6 Gy | 0.190 | 1.000 |
| **42** | Control | 0.746 | 0.911 |
| | 2 Gy | 0.205 | 0.866 |
| | 6 Gy | 0.265 | 0.729 |
| **49** | Control | 0.204 | 0.408 |
| | 2 Gy | 0.205 | 0.264 |
| | 6 Gy | 0.265 | 0.502 |
| **56** | Control | 0.204 | 0.881 |
| | 2 Gy | 0.258 | 0.109 |
| | 6 Gy | 0.156 | 0.326 |
| **63** | Control | 0.435 | 0.180 |
| | 2 Gy | 0.451 | 0.203 |
| | 6 Gy | 0.156 | 0.042 |
| **70** | Control | 0.063 | 0.513 |
| | 2 Gy | 0.211 | 1.000 |
| | 6 Gy | 0.156 | 0.032 |
| **77** | Control | 0.331 | 0.045 |
| | 2 Gy | 0.133 | 0.213 |
| | 6 Gy | 0.156 | 0.022 |
| **84** | Control | 0.827 | 0.042 |
| | 2 Gy | 0.135 | 1.000 |
| | 6 Gy | 0.108 | 0.022 |
| **91** | Control | 1.000 | 0.073 |
| | 2 Gy | 0.025 | 0.086 |
| | 6 Gy | 0.280 | 0.008 |
| **98** | Control | 0.196 | 0.916 |
| | 2 Gy | 0.327 | 0.086 |
| | 6 Gy | 0.169 | 0.026 |
| **105** | Control | 0.341 | 0.751 |
| | 2 Gy | 0.221 | 0.086 |
| | 6 Gy | 0.754 | 0.463 |
| **112** | Control | 0.175 | 0.347 |
| | 2 Gy | 0.221 | 0.027 |
| | 6 Gy | 0.347 | 0.917 |
| **119** | Control | 0.347 | 0.175 |
| | 2 Gy | 0.221 | 0.014 |
| | 6 Gy | 0.754 | 0.347 |

**Table S7.** Values of MMP-1 positive staining (% tumor area) corresponding to IHC staining of the tumors from the different conditions studied (IR doses and cell subpopulations).

| Subpopulation | MMP-1 Positive Staining (% Tumor Area) | | |
|---|---|---|---|
| | Control | 2 Gy | 6 Gy |
| **General** | 42.42 | 43.30 | 36.66 |
| **Positive** | 34.09 | 35.01 | 30.78 |
| **Negative** | 30.04 | 28.36 | 24.26 |

**Table S8.** Values of apoptosis (%) of the MCF-7 (A), MDA-MB-231 (B) and SK-BR-3 (C) cell lines in the general subpopulation and positive subpopulation cultures at 0, 2 and 6 Gy IR doses.

| IR Dose | Apoptosis Values (%) | |
|---|---|---|
| | General Subpopulation | Positive Subpopulation |
| **MCF-7** | | |
| **Control** | 4.250 | 11.15 |
| **2 Gy** | 24.40 | 16.16 |
| **6 Gy** | 91.25 | 13.65 |
| **MDA-MB-231** | | |
| **Control** | 45.50 | 8.400 |
| **2 Gy** | 74.05 | 6.950 |
| **6 Gy** | 68.85 | 13.40 |
| **SK-BR-3** | | |
| **Control** | 7.150 | 4.150 |
| **2 Gy** | 16.10 | 38.45 |
| **6 Gy** | 11.15 | 19.25 |

© 2019 by the authors. Licensee MDPI, Basel, Switzerland. This article is an open access article distributed under the terms and conditions of the Creative Commons Attribution (CC BY) license (http://creativecommons.org/licenses/by/4.0/).
